# *Abiotrophia* spp. and *Staphylococcus epidermidis* Endocarditis Treated with Daptomycin

**DOI:** 10.1100/tsw.2008.95

**Published:** 2008-08-08

**Authors:** E. Bishburg, D. Ghuman, A. Cohen, T. Chan, S. Nalmas

**Affiliations:** ^1^ Division of Infectious Diseases, Newark Beth Israel Medical Center, Newark, NJ, USA; ^2^ Division of Hematology-Oncology, Newark Beth Israel Medical Center, Newark, NJ, USA; ^3^ Division of Pathology, Newark Beth Israel Medical Center, Newark, NJ, USA

**Keywords:** Abiotrophia endocarditis, vancomycin failure, daptomycin treatment of endocarditis

## Abstract

Endocarditis due to *Abiotrophia* spp. occurs in about 5% of endocarditis cases. Most of the cases respond to a combination of penicillin and gentamicin, or vancomycin. We describe a case of *Staphylococcus epidermidis* (CONS) and *Abiotrophia* spp. endocarditis that failed vancomycin treatment, but responded to daptomycin and rifampin.

## CASE REPORT

A 23-year-old male with homozygous sickle cell anemia was admitted with vaso-occlusive pain in his legs and lower back. His past medical history was significant for multiple admissions for painful crisis, frequent catheter infections with *Staphylococcus epidermidis* (CONS), treated with vancomycin.

On admission, the physical exam revealed that the patient was afebrile, pulse 84/min, resp. 16/min, and blood pressure 113/65. Scleral icterus was noted. Heart revealed S1 $S_2$, and a 2/6 systolic ejection murmur was heard at the left sternal border. Porta cath site did not reveal any signs of infection.

Lab studies on admission revealed a WBC count of 21.400 cu/mm, hemoglobin of 8.6 g/dl, hematocrit 27.3%, and reticulocyte count 6.3%. On the third day of admission, the patient developed a temperature of 101.6°F.

Because of the patient's history of previous catheter infections with CONS, intravenous (i.v.) vancomycin was started. Port and peripheral blood cultures grew *Abiotrophia* spp. An M-mode echocardiogram showed thickening of the tricuspid valve and mild tricuspid regurgitation.

The porta cath was removed and a peripherally inserted central catheter (PICC) was placed. Rifampin was added to the regimen and the patient was discharged for home antibiotic therapy. Three weeks after initiation of treatment, the patient developed fever and chills and blood cultures grew CONS. Transesophageal echocardiogram (TEE) showed the tip of the central catheter in the superior vena cava and right atrium with echodensities measuring 1.5 cm around it compatible with vegetations. The PICC line was removed and treatment was continued with vancomycin and rifampin after a new PICC line was inserted.

Three weeks later, the patient started having fever and chills and blood cultures again grew *Abiotrophia* spp. and CONS. Repeat TEE showed thickening of the mitral valve and an echodensity on the anterior leaflet measuring 3–5 mm compatible with vegetation; a catheter was visualized in the right atrium without any vegetations. The patient was diagnosed as having endocarditis, and was switched to i.v. daptomycin 6 mg/kg and continued on rifampin 600mg orally daily.

The patient defervesced rapidly and multiple blood cultures remained negative. Repeat TEE 4 weeks after the start of daptomycin showed resolution of the vegetation on the mitral valve and no vegetation on the catheter or in the right atrium. The patient completed 8 weeks of daptomycin and rifampin treatment. On follow-up visits, the patient remained afebrile.

## MICROBIOLOGY

Blood cultures were processed using a Bactec 9240 machine (Becton Dickinson, Sparks Glencoe, MD). Gram stain of positive blood cultures showed Gram-positive pleomorphic cocci and coccobacilli with some balloon forms. Satellitism around a known strain of *S. aureus* ATCC 29213 in chocolate agar and tryptic soy agar supplemented with 5% sheep blood was observed. These satelliting colonies were stained again and colonies that were closest to the *S. aureus* streak showed normal-appearing Gram-positive cocci in chains and pairs, while the more distant colonies appeared as Gram-variable enlarged pleomorphic coccobacilli. Satellitism was also observed around a 0.001% pyridoxal disk. Based on these characteristics, the organism was determined to be *Abiotrophia* spp. Further workup of this organism failed as the organism became nonviable on subculture.

The identification and susceptibility testing of the CONS was carried out using microscan-dried Gram-positive MIC/Combo (Dade Behring, West Sacramento, CA).

## DISCUSSION

*Abiotrophia* spp., formerly called nutritionally variant streptococci, were originally isolated by Frenkel and Hirsch[1] from patients with endocarditis and otitis media. Bouvet et al.[2] performed DNA-DNA hybridization and named the organism as *Streptococcus adjacens* and *Streptococcus defectivus.* In 1995, Kawamura et al.[3] determined the 16S rRNA sequences of these organisms and placed them in the new genus *Abiotrophia,* as *Abiotrophia defectiva* and *Abiotrophia adjacens*. In 1998, Roggenkamp et al.[4] described a new member of the genus called *A. elegans* from a patient with endocarditis.

*Abiotrophia* spp. colonizes the normal oral, genitourinary, and intestinal flora, and has been isolated from a variety of clinical sources[5]. It is thought to be the etiologic cause of endocarditis in 5% of all cases of endocarditis, but this may be an underestimate due to the difficulties associated with the growth and identification of the organism. More than 30% of *Abiotrophia* spp. are resistant to penicillin (PCN), but sensitive to vancomycin *in vitro*[8]. In a rabbit model of endocarditis, the combination of PCN and gentamicin was more effective than PCN alone. Despite the fact that vancomycin does not exhibit bactericidal activity, it is as effective as PCN and gentamicin, and is considered an alternative treatment in patients who failed a combination of PCN and gentamicin[9,10]

Daptomycin is a cyclic lipopeptide antibiotic, with rapid bactericidal activity against a variety of Gram-positive aerobic organisms[12]. In an endocarditis rabbit model study with *Enterococcus faecium*, daptomycin was present in aortic valve vegetations 30 min after single dose[16]. Daptomycin has recently been approved for use for *S. aureus* endocarditis.

Piper et al. reported the susceptibility of 10 isolates of *Abiotrophia/Granulicatella* spp. to daptomycin with an $MIC_{90}$ of <0.125 µg/ml; one isolate exhibited an MIC value of 2 µg/ml[11].

The clinical course of endocarditis with *Abiotrophia* spp. was reported to be more severe than with other *viridans streptococci* or *enterococci.* The bacteriologic failure rate was 41% despite the use of antibiotics that showed *in vitro* activity. Relapse rate was reported as being 17%. Mortality was reported to be between 17 and 20%, while 27% required a prosthetic valve[6,7,13,14,15].

To our knowledge, our case represents the first case of mitral valve endocarditis due to *Abiotrophia* spp. and CONS successfully treated with daptomycin and rifampin. Our patient failed his initial treatment with vancomycin as evidenced by continuously having positive blood cultures for the same organisms. We could not determine the susceptibility of the *Abiotrophia* spp. isolate to vancomycin or to daptomycin due to the nonviability of this isolate on subculture, but the clinical and microbiologic success of this agent in this case supports its potential benefit in similar clinical situations, and further study of daptomycin and rifampin in endocarditis due to *Abiotrophia* spp. is warranted.
